# The Elder Justice Coalition and Reauthorizing the Elder Justice Act

**DOI:** 10.1093/geroni/igaa057.2548

**Published:** 2020-12-16

**Authors:** Robert Blancato

**Affiliations:** Matz Blancato & Associates, Washington, District of Columbia, United States

## Abstract

The presentation and dialogue will focus on the elder justice advocacy community and their work to secure reauthorization of the Elder Justice Act, which was originally passed as part of the ACA. Both grassroots and DC-based efforts have been employed to move this and other legislation forward and to secure much needed funding for the programs designed to end elder abuse.

